# Sampling Conditions for Conforming Voronoi Meshing by the VoroCrust Algorithm

**DOI:** 10.4230/LIPIcs.SoCG.2018.1

**Published:** 2018-06

**Authors:** Ahmed Abdelkader, Chandrajit L. Bajaj, Mohamed S. Ebeida, Ahmed H. Mahmoud, Scott A. Mitchell, John D. Owens, Ahmad A. Rushdi

**Affiliations:** University of Maryland, College Park MD, USA; University of Texas, Austin TX, USA; Sandia National Laboratories, Albuquerque NM, USA; University of California, Davis CA, USA; Sandia National Laboratories, Albuquerque NM, USA; University of California, Davis CA, USA; University of California, Davis CA, USA

**Keywords:** sampling conditions, surface reconstruction, polyhedral meshing, Voronoi

## Abstract

We study the problem of decomposing a volume bounded by a smooth surface into a collection of Voronoi cells. Unlike the dual problem of conforming Delaunay meshing, a principled solution to this problem for generic smooth surfaces remained elusive. VoroCrust leverages ideas from *α*-shapes and the power crust algorithm to produce unweighted Voronoi cells conforming to the surface, yielding the first provably-correct algorithm for this problem. Given an *ϵ*-sample on the bounding surface, with a weak *σ*-sparsity condition, we work with the balls of radius *δ* times the local feature size centered at each sample. The corners of this union of balls are the Voronoi sites, on both sides of the surface. The facets common to cells on opposite sides reconstruct the surface. For appropriate values of *ϵ*, *σ* and *δ*, we prove that the surface reconstruction is isotopic to the bounding surface. With the surface protected, the enclosed volume can be further decomposed into an isotopic volume mesh of fat Voronoi cells by generating a bounded number of sites in its interior. Compared to state-of-the-art methods based on clipping, VoroCrust cells are full Voronoi cells, with convexity and fatness guarantees. Compared to the power crust algorithm, VoroCrust cells are not filtered, are unweighted, and offer greater flexibility in meshing the enclosed volume by either structured grids or random samples.

## 1 Introduction

Mesh generation is a fundamental problem in computational geometry, geometric modeling, computer graphics, scientific computing and engineering simulations. There has been a growing interest in polyhedral meshes as an alternative to tetrahedral or hex-dominant meshes [48]. Polyhedra are less sensitive to stretching, which enables the representation of complex geometries without excessive refinement. In addition, polyhedral cells have more neighbors even at corners and boundaries, which offers better approximations of gradients and local flow distributions. Even compared to hexahedra, fewer polyhedral cells are needed to achieve a desired accuracy in certain applications. This can be very useful in several numerical methods [18], e.g., finite element [42], finite volume [39], virtual element [17] and Petrov-Galerkin [41]. In particular, the accuracy of a number of important solvers, e.g., the two-point flux approximation for conservation laws [39], greatly benefits from a conforming mesh which is *orthogonal* to its dual as naturally satisfied by Voronoi meshes. Such solvers play a crucial role in hydrology [51], computational fluid dynamics [22] and fracture modeling [20].

VoroCrust is the first provably-correct algorithm for generating a volumetric Voronoi mesh whose boundary conforms to a smooth bounding surface, and with quality guarantees. A conforming volume mesh exhibits two desirable properties *simultaneously*: (1) a decomposition of the enclosed volume, and (2) a reconstruction of the bounding surface.

Conforming Delaunay meshing is well-studied [28], but Voronoi meshing is less mature. A common practical approach to polyhedral meshing is to dualize a tetrahedral mesh and *clip*, i.e., intersect and truncate, each cell by the bounding surface [35,43,47,52,55]. Unfortunately, clipping sacrifices the important properties of convexity and connectedness of cells [35], and may require costly constructive solid geometry operations. Restricting a Voronoi mesh to the surface before *filtering* its dual Delaunay facets is another approach [7, 33, 56], but filtering requires extra checks complicating its implementation and analysis; see also Figure 4. An intuitive approach is to locally mirror the Voronoi sites on either side of the surface [34, 57], but we are not aware of any robust algorithms with approximation guarantees in this category. In contrast to these approaches, VoroCrust is distinguished by its simplicity and robustness at producing true unweighted Voronoi cells, leveraging established libraries, e.g., Voro**++** [50], without modification or special cases.

VoroCrust can be viewed as a principled mirroring technique, which shares a number of key features with the power crust algorithm [13]. The power crust literature [7, 8, 10, 12, 13] developed a rich theory for surface approximation, namely the *ϵ*-sampling paradigm. Recall that the power crust algorithm uses an *ϵ*-sample of unweighted points to place weighted sites, so-called *poles*, near the medial axis of the underlying surface. The surface reconstruction is the collection of facets separating power cells of poles on the inside and outside of the enclosed volume.

Regarding samples and poles as primal-dual constructs, power crust performs a *primal-dual-dual-primal dance*. VoroCrust makes a similar dance where weights are introduced differently; the samples are weighted to define unweighted sites tightly hugging the surface, with the reconstruction arising from their unweighted Voronoi diagram. The key advantage is the freedom to place more sites within the enclosed volume without disrupting the surface reconstruction. This added freedom is essential to the generation of graded meshes; a primary virtue of the proposed algorithm. Another virtue of the algorithm is that all samples appear as vertices in the resulting mesh. While the power crust algorithm does not guarantee that, some variations do so by means of filtering, at the price of the reconstruction no longer being the boundary of power cells [7, 11, 32].

The main construction underlying VoroCrust is a suitable union of balls centered on the bounding surface, as studied in the context of non-uniform approximations [26]. Unions of balls enjoy a wealth of results [15, 24, 37], which enable a variety of algorithms [13, 23, 30].

Similar constructions have been proposed for meshing problems in the applied sciences with heuristic extensions to 3D settings; see [40] and the references therein for a recent example. Aichholzer et al. [6] adopt closely related ideas to construct a union of surface balls using power crust poles for sizing estimation. However, their goal was to produce a coarse homeomorphic surface reconstruction. As in [6], the use of balls and *α*-shapes for surface reconstruction was explored earlier, e.g., ball-pivoting [19, 54], but the connection to Voronoi meshing has been absent. In contrast, VoroCrust aims at a decomposition of the enclosed volume into fat Voronoi cells conforming to an isotopic surface reconstruction with quality guarantees.

In a previous paper [4], we explored the related problem of generating a Voronoi mesh that conforms to restricted classes of piecewise-linear complexes, with more challenging inputs left for future work. The approach adopted in [4] does not use a union of balls and relies instead on similar ideas to those proposed for conforming Delaunay meshing [29,45,49].

**Figure 1:**
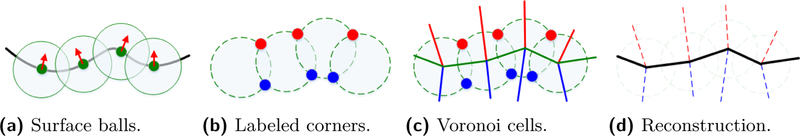
VoroCrust reconstruction, demonstrated on a planar curve.

In this paper, we present a theoretical analysis of an abstract version of the VoroCrust algorithm. This establishes the quality and approximation guarantees of its output for volumes bounded by smooth surfaces. A description of the algorithm we analyze is given next; see Figure 1 for an illustration in 2D, and also our accompanying multimedia contribution [2].

### The abstract VoroCrust algorithm

1. Take as input a sample P on the surface M bounding the volume O.
2. Define a ball *B*_*i*_ centered at each sample *p*_*i*_, with a suitable radius *r*_*i*_, and let $U=\cup_{i}B_{i}$.
3. Initialize the set of sites S with the corner points of ∂U, $S^{↑}$ and $S^{↓}$, on both sides of M.
4. Optionally, generate additional sites $S^{↓↓}$ in the interior of O, and include $S^{↓↓}$ into S.
5. Compute the Voronoi diagram Vor(S) and retain the cells with sites in $S^{↓}\cup S^{↓↓}$ as the volume mesh $\hat{O}$, where the facets between $S^{↑}$ and $S^{↓}$ yield a surface approximation $\hat{M}$.

In this paper, we assume O is a bounded open subset of ${\mathbb{R}}^{3}$, whose boundary M is a closed, bounded and smooth surface. We further assume that P is an *ϵ*-sample, with a weak *σ*-sparsity condition, and *r*_*i*_ is set to *δ* times the local feature size at *p*_*i*_. For appropriate values of *ϵ*, *σ* and *δ*, we prove that $\hat{O}$ and $\hat{M}$ are isotopic to O and M, respectively. We also show that simple techniques for sampling within O, e.g., octree refinement, guarantee an upper bound on the fatness of all cells in $\hat{O}$, as well as the number of samples.

Ultimately, we seek a conforming Voronoi mesher that can handle realistic inputs possibly containing sharp features, can estimate a sizing function and generate samples, and can guarantee the quality of the output mesh. This is the subject of a forthcoming paper [3] which describes the design and implementation of the complete VoroCrust algorithm.

The rest of the paper is organized as follows. Section 2 introduces the key definitions and notation used throughout the paper. Section 3 describes the placement of Voronoi seeds and basic properties of our construction assuming the union of surface balls satisfies a structural property. Section 4 proves this property holds and establishes the desired approximation guarantees under certain conditions on the input sample. Section 5 considers the generation of interior samples and bounds the fatness of all cells in the output mesh. Section 6 concludes the paper with pointers for future work. A number of proofs is deferred to the full version, available online [1]; see also the accompanying multimedia contribution in these proceedings [2].

## 2 Preliminaries

Throughout, standard general position assumptions [38] are made implicitly to simplify the presentation. We use **d**(*p, q*) to denote the Euclidean distance between two points $p,q\in {\mathbb{R}}^{3}$, and B(c,r) to denote the Euclidean ball centered at $c\in {\mathbb{R}}^{3}$ with radius *r*. We proceed to introduce the notation and recall the key definitions used throughout, following those in [13, 26, 37].

### 2.1 Sampling and approximation

We take as input a set of sample points P⊂M. A local scale or *sizing* is used to vary the sample density. Recall that the *medial axis* [13] of M, denoted by A, is the closure of the set of points in ${\mathbb{R}}^{3}$ with more than one closest point on M. Hence, A has one component inside O and another outside. Each point of A is the center of a *medial ball* tangent to M at multiple points. Likewise, each point on M has two tangent medial balls, not necessarily of the same size. The *local feature size* at x∈M is defined as $\text{lfs}\left(x\right)=\inf_{a\in A}\text{d}\left(x,a\right)$. The set P is an *ϵ -sample* [9] if for all x∈M there exists p∈P such that d(x,p)≤ϵ⋅lfs(x).

We desire an approximation of O by a Voronoi mesh $\hat{O}$, where the boundary $\hat{M}$ of $\hat{O}$ approximates M. Recall that two topological spaces are *homotopy-equivalent* [26] if they have the same topology type. A stronger notion of topological equivalence is *homeomorphism*, which holds when there exists a continuous bijection with a continuous inverse from M to $\hat{M}$. The notion of isotopy captures an even stronger type of equivalence for surfaces *embedded* in Euclidean space. Two surfaces M, $\hat{M}\subset {\mathbb{R}}^{3}$ are *isotopic* [16, 25] if there is a continuous mapping $F:M\times \left[0,1\right]\to {\mathbb{R}}^{3}$ such that for each *t* ∈ [0, 1], *F* (·*, t*) is a homeomorphism from M to $\hat{M}$, where *F* (·, 0) is the identity of M and $F\left(M,1\right)=\hat{M}$. To establish that two surfaces are *geometrically close*, the distance between each point on one surface and its closest point on the other surface is required. Such a bound is usually obtained in the course of proving isotopy.

### 2.2 Diagrams and triangulations

The set of points defining a Voronoi diagram are traditionally referred to as *sites* or *seeds*. When approximating a manifold by a set of sample points of varying density, it is helpful to assign weights to the points reflective of their density. In particular, a point *p*_*i*_ with weight *w*_*i*_, can be regarded as a ball *B*_*i*_ with center *p*_*i*_ and radius $r_{i}=\sqrt{w_{i}}$.

Recall that the *power distance* [37] between two points *p*_*i*_*, p*_*j*_ with weights *w*_*i*_*, w*_*j*_ is *π*(*p*_*i*_*, p*_*j*_) = **d**(*p*_*i*_*, p*_*j*_)^2^ −*w*_*i*_ −*w*_*j*_. Unless otherwise noted, points are *unweighted*, having weight equal to zero. There is a natural geometric interpretation of the weight: all points *q* on the boundary of *B*_*i*_ have *π*(*p*_*i*_*, q*) = 0, inside *π*(*p*_*i*_*, q*) *<* 0 and outside *π*(*p*_*i*_*, q*) *>* 0. Given a set of weighted points P, this metric gives rise to a natural decomposition of ${\mathbb{R}}^{3}$ into the *power cells* $V_{i}=\left\{q\in {\mathbb{R}}^{3}|\pi \left(p_{i},q\right)\leq \pi \left(p_{j},q\right)\forall p_{j}\in P\right\}$. The *power diagram* wVor(P) is the cell complex defined by collection of cells *V*_*i*_ for all $p_{i}\in P$.

The nerve [37] of a collection C of sets is defined as N(C)={X⊆C|∩ T≠ϕ}. Observe that N(C) is an abstract simplicial complex because X∈N(C) and Y⊆X imply Y∈N(C). With that, we obtain the *weighted Delaunay triangulation*, or *regular triangulation*, as wDel(P)=N(wVor(P)). Alternatively, wDel(P) can be defined directly as follows. A subset $T\subset {\mathbb{R}}^{d}$, with *d* ≤ 3 and |*T*| ≤ *d*+1 defines a *d*-simplex *σ*_*T*_. Recall that the *orthocenter* [27] of *σ*_*T*_, denoted by *z*_*T*_, is the unique point $q\in {\mathbb{R}}^{d}$ such that *π*(*p*_*i*_*, z*_*T*_) = *π*(*p*_*j*_*, z*_*T*_) for all *p*_*i*_*, p*_*j*_ ∈ *T*; the *orthoradius* of *σ*_*T*_ is equal to *π*(*p, z*_*T*_) for any *p* ∈ *T*. The *Delaunay condition* defines wDel(P) as the set of tetrahedra *σ*_*T*_ with an *empty orthosphere*, meaning *π*(*p*_*i*_*, z*_*T*_ ) ≤ *π*(*p*_*j*_*, z*_*T*_ ) for all *p*_*i*_ ∈ *T* and $p_{j}\in P\backslash T$, where wDel(P) includes all faces of *σ*_*T*_.

There is a natural duality between wDel(P) and wVor(P). For a tetrahedron *σ*_*T*_, the definition of *z*_*T*_ immediately implies *z*_*T*_ is a *power vertex* in wVor(P). Similarly, for each *k*-face *σ*_*S*_ of $\sigma_{T}\in \text{wDel}\left(P\right)$ with S⊆T and *k* + 1 = |*S*|, there exists a dual (3 − *k*)-face $\sigma_{S}^{'}$ in wVor(P) realized as $\cap_{p\in S}V_{p}$. When P is unweighted, the same definitions yield the standard (unweighted) Voronoi diagram Vor(P) and its dual Delaunay triangulation Del(P).

### 2.3 Unions of balls

Let B denote the set of balls corresponding to a set of weighted points P and define the *union of balls* U as ∪B. It is quite useful to capture the structure of U using a combinatorial representation like a simplicial complex [36, 37]. Let *f*_*i*_ denote $V_{i}\cap \partial B_{i}$ and F the collection of all such *f*_*i*_. Observing that $V_{i}\cap B_{j}\subseteq V_{i}\cap B_{i}\forall B_{i}$, $B_{j}\in B$, *f*_*i*_ is equivalently defined as the spherical part of $\partial \left(V_{i}\cap B_{i}\right)$. Consider also the decomposition of U by the cells of wVor(P) into $C\left(B\right)=\left\{V_{i}\cap B_{i}|B_{i}\in B\right\}$. The *weighted α-complex* W(P) is defined as the *geometric realization* of N(C(B)) [37], *i.e.*, $\sigma_{T}\in W$ if $\left\{V_{i}\cap B_{i}|p_{i}\in T\right\}\in N\left(C\left(B\right)\right)$. It is not hard to see that W is a subcomplex of wDel(P).

To see why W is relevant, consider its *underlying space*; we create a collection containing the convex hull of each simplex in W and define the *weighted α-shape* J(P) as the union of this collection. It turns out that the simplices $\sigma_{T}\in W$ contained in ∂J are dual to the faces of ∂U defined as $\cap_{i\in T}f_{i}$. Every point q∈∂U defined by $\cap_{i\in T_{q}}f_{i}$, for $T_{q}\in B$ and *k* + 1 = |*T*_*q*_|, witnesses the existence of $\sigma_{T_{q}}$ in W; the *k*-simplex $\sigma_{T_{q}}$ is said to be *exposed* and ∂J can be defined directly as the collection of all exposed simplices [36]. In particular, the *corners* of ∂U correspond to the facets of ∂J. Moreover, J is homotopy-equivalent to U [37].

The union of balls defined using an *ϵ*-sampling guarantees the approximation of the manifold under suitable conditions on the sampling. Following earlier results on uniform sampling [46], an extension to non-uniform sampling establishes sampling conditions for the isotopic approximation of hypersurfaces and medial axis reconstruction [26].

## 3 Seed placement and surface reconstruction

We determine the location of Voronoi seeds using the union of balls U. The correctness of our reconstruction depends crucially on how sample balls B overlap. Assuming a certain structural property on U, the surface reconstruction is embedded in the dual shape J.

### 3.1 Seeds and guides

Central to the method and analysis are triplets of sample spheres, i.e., boundaries of sample balls, corresponding to a *guide triangle* in wDel(P). The sample spheres associated with the vertices of a guide triangle intersect contributing a pair of *guide points*. The reconstruction consists of Voronoi facets, most of which are guide triangles.

**Figure 2:**
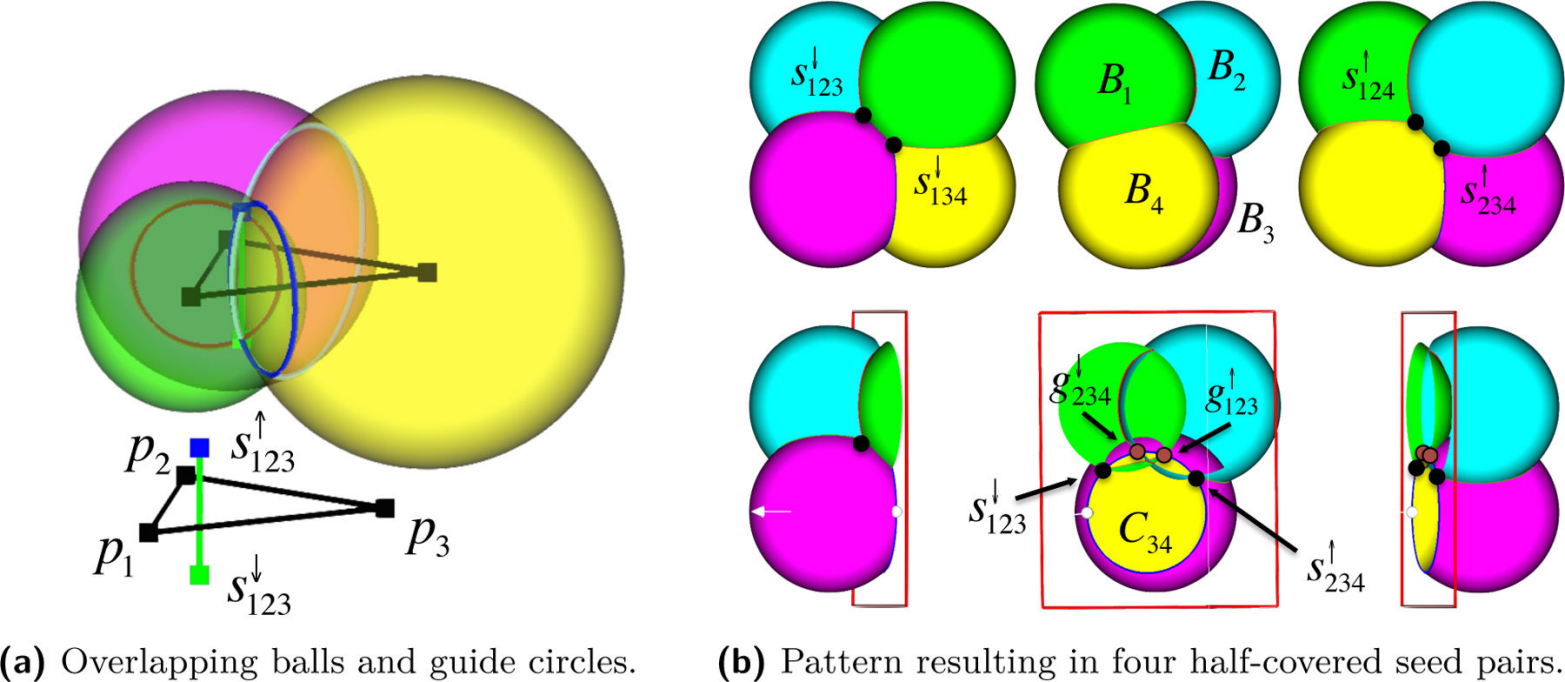
(a) Guide triangle and its dual seed pair. (b) Cutaway view in the plane of circle *C*_34_.

When a triplet of spheres *∂B*_*i*_*, ∂B*_*j*_*, ∂B*_*k*_ intersect at exactly two points, the intersection points are denoted by $g_{ijk}^{↕}=\left\{g_{ijk}^{↑},g_{ijk}^{↓}\right\}$ and called a pair of *guide points* or *guides*; see Figure 2a. The associated *guide triangle t*_*ijk*_ is *dual* to $g_{ijk}^{↕}$. We use arrows to distinguish guides on different sides of the manifold with the *upper* guide *g*^↑^ lying outside O and the *lower* guide *g*^↓^ lying inside. We refer to the edges of guide triangles as *guide edges* $e_{ij}=\bar{p_{i}p_{j}}$. A guide edge *e*_*ij*_ is associated with a dual *guide circle C*_*ij*_ = *∂B*_*i*_ ∩ *∂B*_*j*_, as in Figure 2a.

The Voronoi seeds in $S^{↑}\cup S^{↓}$ are chosen as the subset of guide points that lie on ∂U. A guide point *g* which is not interior to any sample ball is *uncovered* and included as a *seed s* into S; covered guides are not. We denote *uncovered guides* by *s* and *covered guides* by *g*, whenever coverage is known and important. If only one guide point in a pair is covered, then we say the guide pair is *half-covered*. If both guides in a pair are covered, they are ignored. Let $S_{i}=S\cap \partial B_{i}$ denote the seeds on sample sphere *∂B*_*i*_.

**Figure 3:**
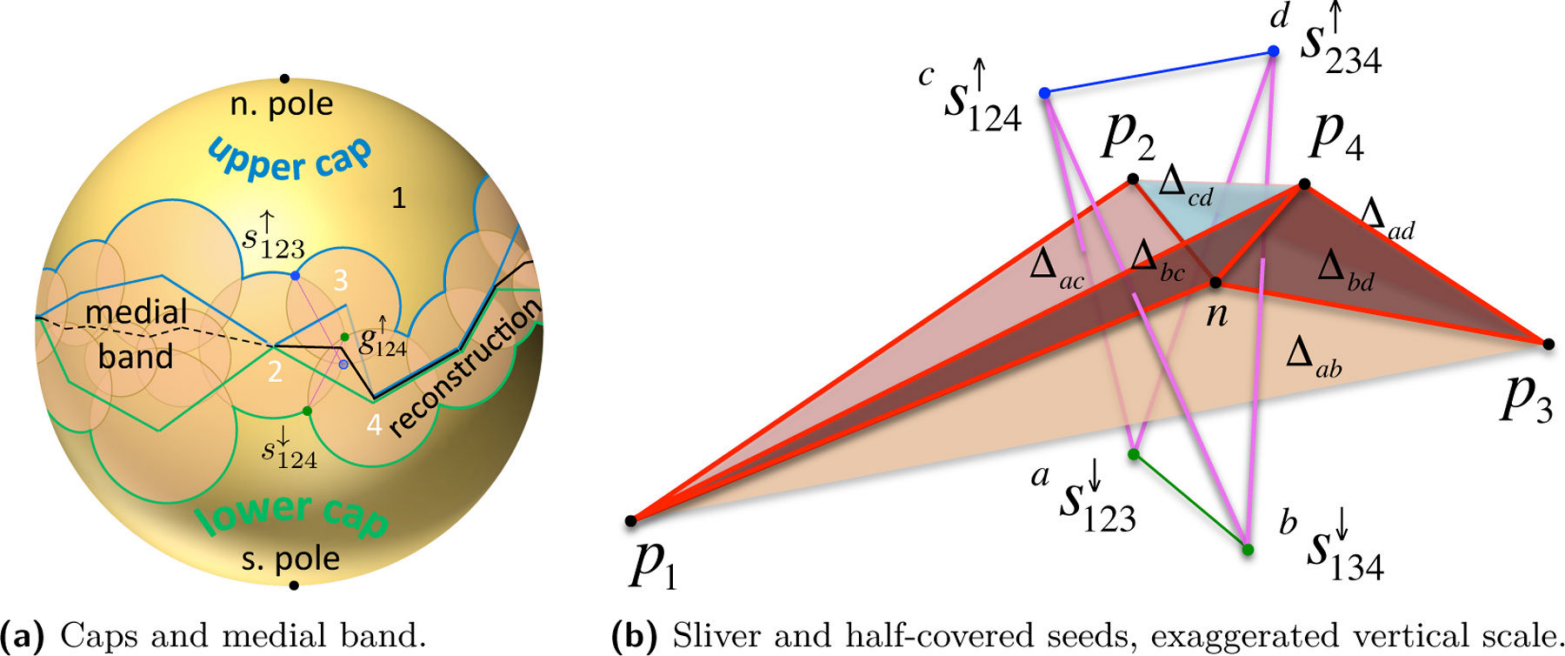
(a) Decomposing the sample sphere *∂B*_1_. (b) Uncovered seeds and reconstruction facets. Let $\tau_{p}\in W\left(P\right)\subseteq \text{wDel}\left(P\right)$ and $\tau_{s}\in \text{Del}\left(S\right)$ denote the tetrahedra connecting the four samples and the four seeds shown, respectively. $s_{123}^{↓}$ and $s_{134}^{↓}$ are the uncovered lower guide seeds, with $g_{123}^{↑}$ and $g_{134}^{↑}$ covered. The uncovered upper guide seeds are $s_{124}^{↑}$ and $s_{234}^{↑}$, with $g_{124}^{↓}$ and $g_{234}^{↓}$ covered. $\Delta_{ac}$ is the Voronoi facet dual to the Delaunay edge between ${\;}^{a}s_{123}^{↓}$ and ${\;}^{c}s_{124}^{↑}$, etc. Voronoi facets dual to magenta edges are in the reconstructed surface; those dual to green and blue edges are not. *n* is the circumcenter of *τ*_*s*_ and appears as a Voronoi vertex in Vor(S) and a *Steiner vertex* in the surface reconstruction. In general, *n* is not the orthocenter of the sliver *τ*_*p*_.

As each guide triangle *t*_*ijk*_ is associated with at least one dual seed *s*_*ijk*_, the seed witnesses its inclusion in W and *t*_*ijk*_ is exposed. Hence, *t*_*ijk*_ belongs to ∂J as well. When such *t*_*ijk*_ is dual to a single seeds *s*_*ijk*_ it bounds the interior of J, i.e., it is a face of a *regular component* of J; in the simplest and most common case, *t*_*ijk*_ is a facet of a tetrahedron as shown in Figure 3b. When *t*_*ijk*_ is dual to a pair of seeds $s_{ijk}^{↕}$, it does not bound the interior of J and is called a *singular face* of ∂J. All singular faces of ∂J appear in the reconstructed surface.

### 3.2 Disk caps

We describe the structural property required on U along with the consequences exploited by VoroCrust for surface reconstruction. This is partially motivated by the requirement that all sample points on the surface appear as vertices in the output Voronoi mesh.

We define the subset of *∂B*_*i*_ inside other balls as the *medial band* and say it is *covered*. Let the caps $K_{i}^{↑}$ and $K_{i}^{↓}$ be the complement of the medial band in the interior and exterior of O, respectively. Letting $n_{p_{i}}$ be the normal line through *p*_*i*_ perpendicular to M, the two intersection points $n_{p_{i}}\cap \partial B_{i}$ are called the *poles* of *B*_*i*_. See Figure 3a.

We require that U satisfies the following structural property: each *∂B*_*i*_ has *disk caps*, meaning the medial band is a *topological annulus* and the two caps contain the poles and are *topological disks*. In other words, each *B*_*i*_ contributes one connected component to each side of ∂U. As shown in Figure 3a, all seeds in $S_{i}^{↑}$ and $S_{i}^{↓}$ lie on $\partial K_{i}^{↑}$ and $\partial K_{i}^{↓}$, respectively, along the arcs where other sample balls intersect *∂B*_*i*_. In Section 4, we establish sufficient sampling conditions to ensure U satisfies this property. In particular, we will show that both poles of each *B*_*i*_ lie on ∂U.

The importance of disk caps is made clear by the following observation. The requirement that all sample points appear as Voronoi vertices in $\hat{M}$ follows as a corollary.

**Observation 1** (Three upper/lower seeds). *If ∂B*_*i*_ *has* disk caps*, then each of* $\partial K_{i}^{↑}$ *and* $\partial K_{i}^{↓}$ *has at least three seeds and the seeds on ∂B*_*i*_ *are not all coplanar.*

**Proof.** Every sphere $S_{j\neq i}$ covers strictly less than one hemisphere of *∂B*_*i*_ because the poles are uncovered. Hence, each cap is composed of at least three arcs connecting at least three upper seeds $S_{i}^{↑}\subset \partial K_{i}^{↑}$ and three lower seeds $S_{i}^{↓}\subset \partial K_{i}^{↓}$. Further, any hemisphere through the poles contains at least one upper and one lower seed. It follows that the set of seeds $S_{i}=S_{i}^{↑}\cup S_{i}^{↓}$ is not coplanar.

**Corollary 2** (Sample reconstruction)**.** *If ∂B*_*i*_ *has disk caps, then p*_*i*_ *is a vertex in* $\hat{M}$.

**Proof.** By Observation 1, the sample is equidistant to at least four seeds which are not all coplanar. It follows that the sample appears as a vertex in the Voronoi diagram and not in the relative interior of a facet or an edge. Being a common vertex to at least one interior and one exterior Voronoi seed, VoroCrust retains this vertex in its output reconstruction.

### 3.3 Sandwiching the reconstruction in the dual shape of U

Triangulations of smooth surfaces embedded in ${\mathbb{R}}^{3}$ can have half-covered guides pairs, with one guide covered by the ball of a fourth sample not in the guide triangle dual to the guide pair. The tetrahedron formed by the three samples of the guide triangle plus the fourth covering sample is a *sliver*, i.e., the four samples lie almost uniformly around the equator of a sphere. In this case we do not reconstruct the guide triangle, and also do not reconstruct some guide edges. We show that the reconstructed surface $\hat{M}$ lies entirely within the region of space bounded by guide triangles, i.e., the *α*-shape of P, as stated in the following theorem.

**Theorem 3** (Sandwiching)**.** *If all sample balls have disk caps, then* $\hat{M}\subseteq J\left(P\right)$.

**Figure 4:**
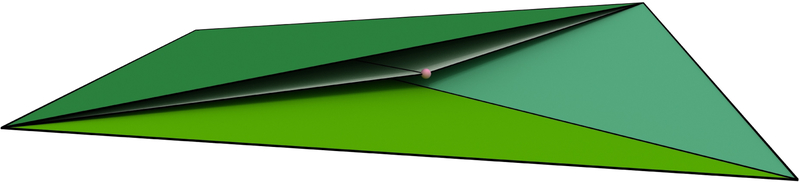
Cutaway view of a sliver tetrahedron $\tau_{p}\in W\left(P\right)\subseteq \text{wDel}\left(P\right)$, drawn to scale. Half-covered guides give rise to the Steiner vertex (pink), which results in a surface reconstruction using four facets (only two are shown) sandwiched within *τ*_*p*_. In contrast, filtering wDel(P) chooses two of the four facets of *τ*_*p*_, either the bottom two, or the top two (only one is shown).

The simple case of a single isolated sliver tetrahedron is illustrated in Figures 3b, 4 and 2b. A sliver has a pair of lower guide triangles and a pair of upper guide triangles. For instance, *t*_124_ and *t*_234_ are the pair of upper triangles in Figure 3b. In such a tetrahedron, there is an edge between each pair of samples corresponding to a non-empty circle of intersection between sample balls, like the circles in Figure 2a. For this circle, the arcs covered by the two other sample balls of the sliver overlap, so each of these balls contributes exactly one uncovered seed, rather than two. In this way the upper guides for the upper triangles are uncovered, but their lower guides are covered; also only the lower guides of the lower triangles are uncovered. The proof of Theorem 3 follows by analyzing the Voronoi cells of the seed points located on the overlapping sample balls and is deferred to Appendix A [1]. Alternatively, Theorem 3 can be seen as a consequence of Theorem 2 in [15].

## 4 Sampling conditions and approximation guarantees

We take as input a set of points P sampled from the bounding surface M such that P is an *ϵ*-sample, with *ϵ* ≤ 1*/*500. We require that P satisfies the following sparsity condition: for any two points *p*_*i*_*, p*_*j*_ ∈ *P*, $\text{lfs}\left(p_{i}\right)\geq \text{lfs}\left(p_{j}\right)\Rightarrow \text{d}\left(p_{i},p_{j}\right)\geq \sigma \epsilon \text{lfs}\left(p_{j}\right)$, with *σ* ≥ 3*/*4.

Such a sampling P can be obtained by known algorithms. Given a suitable representation of M, the algorithm in [21] computes a loose *ϵ*′-sample *E* which is a *ϵ*′(1+8.5*ϵ*′)-sample. More specifically, whenever the algorithm inserts a new sample *p* into the set *E*, $\text{d}\left(p,E\right)\geq \epsilon^{\prime }\text{lfs}\left(p\right)$. To obtain *E* as an *ϵ*-sample, we set $\epsilon^{\prime }\left(\epsilon \right)=\left(\sqrt{34\epsilon +1}-1\right)/17$. Observing that $3\epsilon /4\leq \epsilon^{\prime }\left(\epsilon \right)$ for *ϵ* ≤ 1*/*500, the returned *ϵ*-sample satisfies our required sparsity condition with *σ* ≥ 3*/*4.

We start by adapting Theorem 6.2 and Lemma 6.4 from [26] to the setting just described. For $x\in {\mathbb{R}}^{3}\backslash M$, let $\Gamma \left(x\right)=\text{d}\left(x,\tilde{x}\right)/\text{lfs}\left(\tilde{x}\right)$, where $\tilde{x}$ is the closest point to *x* on M.

**Corollary 4.** *For an ϵ -sample* P, *with ϵ* ≤ 1*/*20*, the union of balls* U *with δ* = 2*ϵ satisfies:*

1. M *is a deformation retract of* U,
2. ∂U *contains two connected components, each isotopic to* M,
3. $\Gamma^{-1}\left(\left[0,a^{\prime }\right]\right)\subset U\subset \Gamma^{-1}\left(\left[0,b^{\prime }\right]\right)$, *where a*′ = *ϵ* − 2*ϵ*^2^ *and b*′ ≤ 2.5*ϵ.*

**Proof.** Theorem 6.2 from [26] is stated for balls with radii within [*a, b*] times the lfs. We set *a* = *b* = *δ* and use *ϵ* ≤ 1*/*20 to simplify fractions. This yields the above expressions for *a*′ = (1 – *ϵ*)*δ* − *ϵ* and *b*′ = *δ/*(1 − 2*δ*). The general condition requires (1 – *a*′)^2^ + (*b*′ − *a*′+ *δ*(1 + 2*b*′ − *a*′)*/*(1 − *δ*))^2^ < 1, as we assume no noise. Plugging in the values of *a*′ and *b*′, we verify that the inequality holds for the chosen range of *ϵ*.

Furthermore, we require that each ball $B_{i}\in B$ contributes one facet to each side of ∂U . Our sampling conditions ensure that both poles are outside any ball $B_{j}\in B$.

**Lemma 5** (Disk caps)**.** *All balls in* B *have disk caps for ϵ* ≤ 0.066*, δ* = 2*ϵ and σ* ≥ 3*/*2.

**Proof.** Fix a sample *p*_*i*_ and let *x* be one of the poles of *B*_*i*_ and $B_{x}=B\left(c,\text{lfs}\left(p_{i}\right)\right)$ the tangent ball at *p*_*i*_ with *x* ∈ *B*_*x*_. Letting *p*_*j*_ be the closest sample to *x* in *P* \ {*p*_*i*_}, we assume the worst case where lfs(*p*_*j*_) ≥ lfs(*p*_*i*_) and *p*_*j*_ lies on *∂B*_*x*_. To simplify the calculations, take lfs(*p*_*i*_) = 1 and let *ℓ* denote **d**(*p*_*i*_*, p*_*j*_). As lfs is 1-Lipschitz, we get lfs(*p*_*j*_) ≤ 1 + *ℓ*. By the law of cosines, **d**(*p*_*j*_*, x*)^2^ = **d**(*p*_*i*_*, p*_*j*_)^2^ + **d**(*p*_*i*_*, x*)^2^ − 2**d**(*p*_*i*_*, p*_*j*_)**d**(*p*_*i*_*, x*) cos(*ϕ*), where $\phi =∠p_{j}p_{i}c$. Letting $\theta =∠p_{i}cp_{j}$, observe that cos(*ϕ*) = sin(*θ/*2) = *ℓ/*2. To enforce $x\notin B_{j}$, we require **d**(*p*_*j*_*, x*) *> δ*lfs(*p*_*j*_), which is equivalent to $l^{2}+\delta^{2}-\delta l^{2}>\delta^{2}{\left(1+l\right)}^{2}$. Simplifying, we get $l>2\delta^{2}/\left(1-\delta -\delta^{2}\right)$ where sparsity guarantees *ℓ > σϵ*. Setting $\sigma \epsilon >2\delta^{2}/\left(1-\delta -\delta^{2}\right)$ we obtain $4\sigma \epsilon^{2}+\left(8+2\sigma \right)\epsilon -\sigma <0$, which requires *ϵ<* 0.066 when *σ* ≥ 3*/*4.

Theorem 4 together with Theorem 5 imply that each *∂B*_*i*_ is decomposed into a covered region $\partial B_{i}\cap \cup_{j\neq i}B_{j}$, the *medial band*, and two uncovered caps $\partial B_{i}\backslash \cup_{j\neq i}B_{j}$, each containing one pole. Recalling that seeds arise as pairs of intersection points between the boundaries of such balls, we show that seeds can be classified correctly as either inside or outside M.

**Corollary 6.** *If a seed pair lies on the same side of* M, *then at least one seed is covered.*

**Proof.** Fix such a seed pair *∂B*_*i*_ ∩ *∂B*_*j*_ ∩ *∂B*_*k*_ and recall that $M\cap \partial B_{i}$ is contained in the medial band on *∂B*_*i*_. Now, assume for contradiction that both seeds are uncovered and lie on the same side of M. It follows that *B*_*j*_ ∩ *B*_*k*_ intersects *B*_*i*_ away from its medial band, a contradiction to Theorem 4.

Theorem 4 guarantees that the medial band of *B*_*i*_ is a superset of $\Gamma^{-1}\left(\left[0,a^{\prime }\right]\right)\cap \partial B_{i}$, which means that all seeds *s*_*ijk*_ are at least $a^{\prime }\text{lfs}\left({\tilde{s}}_{ijk}\right)$ away from M. It will be useful to bound the elevation of such seeds above $T_{p_{i}}$, the *tangent plane* to M at *p*_*i*_.

**Lemma 7.** *For a seed s* ∈ *∂B*_*i*_, $\theta_{s}=∠sp_{i}s^{\prime }\geq 29.34^{\circ }$ *and* $\theta_{s}>\frac{1}{2}-5\epsilon$, *where s*′ *is the projection of s on* $T_{p_{i}}$, implying $\text{d}\left(s,s^{\prime }\right)\geq h_{s}^{⊥}\delta lfs\left(p_{i}\right)$, *with* $h_{s}^{⊥}>0.46$ and $h_{s}^{⊥}>\frac{1}{2}-5\epsilon$.

**Figure 5:**
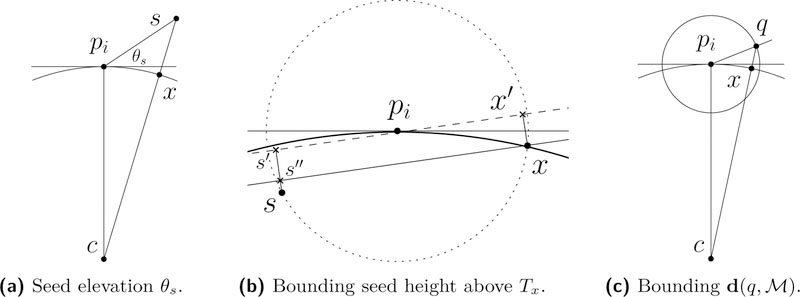
Constructions used for (a) Theorem 7, (b) Theorem 12 and (c) Theorem 13.

**Proof.** Let lfs(*p*_*i*_) = 1 and $B_{s}=B\left(c,1\right)$ be the tangent ball at *p*_*i*_ with $s\notin B_{s}$; see Figure 5a. Observe that d(s,M)≤d(s,x), where $x=\bar{sc}\cap \partial B_{s}$. By the law of cosines, $\text{d}{\left(s,c\right)}^{2}=\text{d}{\left(p_{i},c\right)}^{2}+\text{d}{\left(p_{i},s\right)}^{2}-2\text{d}\left(p_{i},c\right)\text{d}\left(p_{i},s\right)\cos\left(\pi /2+\theta_{s}\right)=1+\delta^{2}+2\delta \sin\left(\theta_{s}\right)$. We may write^2^ $\text{d}\left(s,c\right)\leq 1+\delta^{2}/2+\delta \sin\left(\theta_{s}\right)$. It follows that $\text{d}\left(s,x\right)\leq \delta^{2}/2+\delta \sin\left(\theta_{s}\right)$. As lfs is 1-Lipschitz and $\text{d}\left(p_{i},x\right)\leq \delta$, we get 1−δ≤lfs(x)≤1+δ. There must exist a sample *p*_*j*_ such that $\text{d}\left(x,p_{j}\right)\leq \epsilon \text{lfs}\left(x\right)\leq \epsilon \left(1+\delta \right)$. Similarly, $\text{lfs}\left(p_{j}\right)\geq \left(1-\epsilon \left(1+\delta \right)\right)\left(1-\delta \right)$. By the triangle inequality, $\text{d}\left(s,p_{j}\right)\leq \text{d}\left(s,x\right)+\text{d}\left(x,p_{j}\right)\leq \delta^{2}/2+\delta \sin\left(\theta_{s}\right)+\epsilon \left(1+\delta \right)$. Setting $\text{d}\left(s,p_{j}\right)<\delta \left(1-\delta \right)\left(1-\epsilon \left(1+\delta \right)\right)$ implies $\text{d}\left(s,p_{j}\right)<\delta \text{lfs}\left(p_{j}\right)$, which shows that for small values of *θ*_*s*_, *s* cannot be a seed and $p_{j}\neq p_{i}$. Substituting *δ* = 2*ϵ*, we get $\theta_{s}\geq \sin^{-1}\left(2\epsilon^{3}-5\epsilon +1/2\right)\geq 29.34^{\circ }$ and $\theta_{s}>1/2-5\epsilon$.

We make frequent use of the following bound on the distance between related samples.

**Claim 8.** *If* $B_{i}\cap B_{j}\neq \phi$*, then* $d\left(p_{i},p_{j}\right)\in \left[\kappa_{\epsilon },\kappa \delta \right]\cdot lfs\left(p_{i}\right)$, *with κ* = 2*/*(1 − *δ*) *and* $\kappa_{\epsilon }=\sigma \epsilon /\left(1+\sigma \epsilon \right)$.

**Proof.** The upper bound comes from **d**(*p*_*i*_*, p*_*j*_) ≤ *r*_*i*_ + *r*_*j*_ and lfs(*p*_*j*_) ≤ lfs(*p*_*i*_) + **d**(*p*_*i*_*, d*_*j*_) by 1-Lipschitz, and the lower bound from lfs(*p*_*i*_) − **d**(*p*_*i*_*, d*_*j*_) ≤ lfs(*p*_*j*_) and the sparsity.

Bounding the circumradii is the culprit behind why we need such small values of *ϵ*.

**Lemma 9.** *The circumradius of a guide triangle t*_*ijk*_ *is at most* $\varrho_{f}\cdot \delta lfs\left(p_{i}\right)$, *where* $\varrho_{f}<1.38$, *and at most* ${\bar{\varrho }}_{f}\cdot d\left(p_{i},p_{j}\right)$ where ${\bar{\varrho }}_{f}<3.68$.

**Proof.** Let *p*_*i*_ and *p*_*j*_ be the triangle vertices with the smallest and largest lfs values, respectively. From Claim 8, we get **d**(*p*_*i*_*, p*_*j*_) ≤ *κδ*lfs(*p*_*i*_). It follows that lfs(*p*_*j*_) ≤ (1+*κδ*)lfs(*p*_*i*_). As *t*_*ijk*_ is a guide triangle, we know that it has a pair of intersection points *∂B*_*i*_ ∩ *∂B*_*j*_ ∩ *∂B*_*k*_. Clearly, the seed is no farther than *δ*lfs(*p*_*j*_) from any vertex of *t*_*ijk*_ and the orthoradius of *t*_*ijk*_ cannot be bigger than this distance.

Recall that the weight *w*_*i*_ associated with *p*_*i*_ is *δ*^2^lfs(*p*_*i*_)^2^. We shift the weights of all the vertices of *t*_*ijk*_ by the lowest weight *w*_*i*_, which does not change the orthocenter. With that $w_{j}-w_{i}=\delta^{2}\left(\text{lfs}{\left(p_{j}\right)}^{2}-\text{lfs}{\left(p_{i}\right)}^{2}\right)\leq \delta^{2}\text{lfs}{\left(p_{i}\right)}^{2}\left({\left(1+\kappa \delta \right)}^{2}-1\right)=\kappa \delta^{3}\text{lfs}{\left(p_{i}\right)}^{2}\left(\kappa \delta +2\right)$ . On the other hand, sparsity ensures that the closest vertex in *t*_*ijk*_ to *p*_*j*_ is at distance at least $N\left(p_{j}\right)\geq \sigma \epsilon \text{lfs}\left(p_{j}\right)\geq \sigma \epsilon \left(1-\kappa \delta \right)\text{lfs}\left(p_{i}\right)$. Ensuring $\alpha^{2}\leq \left(w_{j}-w_{i}\right)/N{\left(p_{i}\right)}^{2}\leq \kappa \delta^{3}\left(2+\kappa \delta \right)/\left(\sigma^{2}\epsilon^{2}{\left(1-\kappa \delta \right)}^{2}\right)\leq 1/4$ suffices to bound the circumradius of *t*_*ijk*_ by $c_{rad}=1/\sqrt{1-4\alpha^{2}}$ times its orthoradius, as required by Claim 4 in [27]. Substituting *δ* = 2*ϵ* and *σ* ≥ 3*/*4 we get *α*^2^ ≤ 78.97*ϵ*, which corresponds to *c*_*rad*_ *<* 1.37. It follows that the circumradius is at most $c_{rad}\delta \text{lfs}\left(p_{j}\right)\leq c_{rad}\left(1+\kappa \delta \right)\delta \text{lfs}\left(p_{i}\right)<1.38\delta \text{lfs}\left(p_{i}\right)$.

For the second statement, observe that $\text{lfs}\left(p_{i}\right)\geq \left(1-\kappa \delta \right)\text{lfs}\left(p_{j}\right)$ and the sparsity condition ensures that the shortest edge length is at least $\sigma \epsilon \text{lfs}\left(p_{i}\right)\geq \sigma \epsilon \left(1-\kappa \delta \right)\text{lfs}\left(p_{j}\right)$. It follows that the circumradius is at most $\frac{\delta c_{rad}}{\sigma \epsilon \left(1-\kappa \delta \right)}<3.68$ times the length of any edge of *t*_*ijk*_.

Given the bound on the circumradii, we are able to bound the deviation of normals.

**Lemma 10.** *If t*_*ijk*_ *is a guide triangle, then (1)* ${∠}_{a}\left(n_{p_{i}},n_{p_{j}}\right)\leq \eta_{s}\delta <0.47^{\circ }$, *with η*_*s*_ *<* 2.03*, and (2) ${∠}_{a}\left(n_{t},n_{p_{i}}\right)\leq \eta_{t}\delta <1.52^{\circ }$, with η*_*t*_ *<* 6.6*, where $n_{p_{i}}$ is the line normal to M at p*_*i*_ *and n*_*t*_ *is the normal to t*_*ijk*_*. In particular, t*_*ijk*_ *makes an angle at most η*_*t*_*δ with* $T_{p_{i}}$.

**Proof.** Claim 8 implies **d**(*p*_*i*_*, p*_*j*_) ≤ *κδ*lfs(*p*_*i*_) and (1) follows from the Normal Variation Lemma [14] with *ρ* = *κδ <* 1*/*3 yielding ${∠}_{a}\left(n_{p_{i}},n_{p_{j}}\right)\leq \kappa \delta /\left(1-\kappa \delta \right)$. Letting *R*_*t*_ denote the circumradius of *t*, Theorem 9 implies that the $R_{t}\leq \varrho_{f}\cdot \delta \text{lfs}\left(p_{i}\right)\leq \text{lfs}\left(p_{i}\right)/\sqrt{2}$ and the Triangle Normal Lemma [31] implies ${∠}_{a}\left(n_{p^{*}},n_{t}\right)<4.57\delta <1.05^{\circ }$, where *p*^∗^ is the vertex of *t* subtending a maximal angle in *t*. Hence, ${∠}_{a}\left(n_{p_{i}},n_{t}\right)\leq {∠}_{a}\left(n_{p_{i}},n_{p^{*}}\right)+{∠}_{a}\left(n_{p^{*}},n_{t}\right)$.

Towards establishing homeomorphism, the next lemma on the monotonicity of distance to the nearest seed is critical. First, we show that the nearest seeds to any surface point x∈M are generated by nearby samples.

**Lemma 11.** *The nearest seed to* x∈M *lies on some ∂B*_*i*_ *where* $d\left(x,p_{i}\right)\leq 5.03\cdot \epsilon lfs\left(x\right)$. *Consequently*, $d\left(x,p_{i}\right)\leq 5.08\cdot \epsilon lfs\left(p_{i}\right)$.

**Proof.** In an *ϵ*-sampling, there exists a *p*_*a*_ such that **d**(*x, p*_*a*_) ≤ *ϵ*lfs(*x*), where lfs(*p*_*a*_) ≤ (1 + *ϵ*)lfs(*x*). The sampling conditions also guarantee that there exists at least one seed *s*_*a*_ on *∂B*_*a*_. By the triangle inequality, we get that $\text{d}\left(x,s_{a}\right)\leq \text{d}\left(x,p_{a}\right)+\text{d}\left(p_{a},s_{a}\right)\leq \epsilon \text{lfs}\left(x\right)+\delta \text{lfs}\left(p_{a}\right)\leq \epsilon \left(1+2\left(1+\epsilon \right)\right)\text{lfs}\left(x\right)=\epsilon \left(2\epsilon +3\right)\text{lfs}\left(x\right)$.

We aim to bound *ℓ* to ensure ∀*p*_*i*_ s.t. $\text{d}\left(x,p_{i}\right)=l\cdot \epsilon \text{lfs}\left(x\right)$, the nearest seed to *x* cannot lie on *B*_*i*_. Note that in this case, $\left(1-l\epsilon \right)\text{lfs}\left(x\right)\leq \text{lfs}\left(p_{i}\right)\leq \left(1+l\epsilon \right)\text{lfs}\left(x\right)$. Let *s*_*i*_ be any seed on *B*_*i*_. It follows that $\text{d}\left(x,s_{i}\right)\geq \text{d}\left(x,p_{i}\right)-\text{d}\left(p_{i},s_{i}\right)\geq l\cdot \epsilon \text{lfs}\left(x\right)-2\epsilon \text{lfs}\left(p_{i}\right)\geq \epsilon \left(\left(1-2\epsilon \right)l-2\right)\text{lfs}\left(x\right)$.

Setting ϵ((1−2ϵ)l−2)lfs(x)≥ϵ(2ϵ+3)lfs(x) suffices to ensure $\text{d}\left(x,s_{i}\right)\geq \text{d}\left(x,s_{a}\right)$, and we get l≥(2ϵ+5)/(1−2ϵ). Conversely, if the nearest seed to *x* lies on *B*_*i*_, it must be case that $\text{d}\left(x,p_{i}\right)\leq l\epsilon \text{lfs}\left(x\right)$. We verify that lϵ=ϵ(2ϵ+5)/(1−2ϵ)<1 for any *ϵ <* 0.13. It follows that $\text{d}\left(x,p_{j}\right)\leq l\epsilon /\left(1-l\epsilon \right)\text{lfs}\left(p_{i}\right)$.

**Lemma 12.** *For any normal segment N*_*x*_ *issued from* x∈M, *the distance to* $S^{↑}$ *is either strictly increasing or strictly decreasing along $\Gamma^{-1}\left(\left[0,0.96\epsilon \right]\right)\cap N_{x}$. The same holds for $S^{↓}$.*

**Proof.** Let *n*_*x*_ be the outward normal and *T*_*x*_ be the tangent plane to M at *x*. By Theorem 11, the nearest seeds to *x* are generated by nearby samples. Fix one such nearby sample *p*_*i*_. For all possible locations of a seed $s\in S^{↑}\cap \partial B_{i}$, we will show a sufficiently large lower bound on $\langle s-s^{\prime \prime },n_{x}\rangle$, where *s′′* the projection of *s* onto *T*_*x*_.

Take lfs(*p*_*i*_) = 1 and let $B_{s}=B\left(c,1\right)$ be the tangent ball to M at *p*_*i*_ with *s* ∈ *B*_*s*_. Let *A* be the plane containing {*p*_*i*_*, s, x*}. Assume in the worst case that $A⊥T_{p_{i}}$ and *x* is as far as possible from *p*_*i*_ on $\partial B_{s}\cap T_{p_{i}}$. By Theorem 11, **d**(*p*_*i*_*, x*) ≤ 5.08*ϵ* and it follows that $\theta_{x}=∠\left(n_{x},n_{p_{i}}\right)\leq 5.08\epsilon /\left(1-5.08\epsilon \right)\leq 5.14\epsilon$. This means that *T*_*x*_ is confined within a (*π/*2 − *θ*_*x*_)-cocone centered at *x*. Assume in the worst case that *n*_*x*_ is parallel to *A* and *T*_*x*_ is tilted to minimize **d**(*s, s′′*); see Figure 5b.

Let $T_{x}^{'}$ be a translation of *T*_*x*_ such that $p_{i}\in T_{x}^{'}$ and denote by *x*′ and *s*′ the projections of *x* and *s*, respectively, onto $T_{x}^{'}$. Observe that $T_{x}^{'}$ makes an angle *θ*_*x*_ with $T_{p_{i}}$. From the isosceles triangle $\Delta p_{i}cx$, we get that $\theta_{x}^{'}\leq 1/2∠p_{i}cx=\sin^{-1}5.08\epsilon /2\leq 2.54\epsilon$. Now, consider $\Delta p_{i}xx^{\prime }$ and let $\phi =∠xp_{i}x^{\prime }$. We have that $\phi =\theta_{x}+\theta_{x}^{'}\leq 2.54\epsilon +\delta /\left(1-\delta \right)\leq 4.55\epsilon$. Hence, sin(*ϕ*) ≤ 4.55*ϵ* and **d**(*x, x*′) ≤ 5.08*ϵ* sin(*ϕ*) ≤ 0.05*ϵ*. On the other hand, we have that $∠sp_{i}s^{\prime }=\psi \geq \theta_{s}-\theta_{x}$ and $\text{d}\left(s,s^{\prime }\right)\geq \delta \sin\psi$, where *θ*_*s*_ ≥ 1*/*2−5*ϵ* by Theorem 7. Simplifying we get sin(ψ)≥1/2−10.08ϵ. The proof follows by evaluating $\text{d}\left(s,s^{\prime \prime }\right)=\text{d}\left(s,s^{\prime }\right)-\text{d}\left(x,x^{\prime }\right)$.

**Theorem 13.** *For every* x∈M with closest point $q\in \hat{M}$ and for every $q\in \hat{M}$ with closest point x∈M, we have $\left\|xq\right\|<h_{t}\cdot \epsilon^{2}lfs\left(x\right)$, *where h*_*t*_ *<* 30.52. *For ϵ* < 1/500, $h_{t}\cdot \epsilon^{2}<0.0002$. *Moreover, the restriction of the mapping π to $\hat{M}$ is a homeomorphism and $\hat{M}$ and M are ambient isotopic. Consequently, $\hat{O}$ is ambient isotopic to O as well.*

**Proof.** Fix a sample $p_{i}\in P$ and a surface point $x\in M\cap B_{i}$. We consider two cocones centered at *x*: a *p*-cocone contains all nearby surface points and a *q*-cocone contains all guide triangles incident at *p*_*i*_. By Theorem 3, all reconstruction facets generated by seeds on *B*_*i*_ are sandwiched in the *q*-cocone.

Theorem 10 readily provides a bound on the q-cocone angle as *γ* ≤ *η*_*t*_*δ*. In addition, since **d**(*p*_*i*_*, x*) ≤ *δ*lfs(*p*_*i*_), we can bound the *p*-cocone angle as *θ* ≤ 2 sin^−1^ (*δ/*2) by Lemma 2 in [7]. We utilize a mixed *pq*-cocone with angle *ω* = *γ/*2 + *θ/*2, obtained by gluing the lower half of the *p*-cocone with the upper half of the *q*-cocone.

Let $q\in \hat{M}$ and consider its closest point x∈M. Again, fix $p_{i}\in P$ such that *x* ∈ *B*_*i*_; see Figure 5c. By sandwiching, we know that any ray through *q* intersects at least one guide triangle, in some point *y*, after passing through *x*. Let us assume the worst case that *y* lies on the upper boundary of the *pq*-cocone. Then, $\text{d}\left(q,x\right)\leq \text{d}\left(y,y^{\prime }\right)=h=\delta \sin\left(\omega \right)\text{lfs}\left(p_{i}\right)$, where *y*′ is the closest point on the lower boundary of the *pq*-cocone point to *q*. We also have that, $\text{d}\left(p_{i},x\right)\leq \cos\left(\omega \right)\delta \text{lfs}\left(p_{i}\right)$, and since lfs is 1-Lipschitz, $\text{lfs}\left(p_{i}\right)\leq \text{lfs}\left(x\right)/\left(1-\delta \right)$. Simplifying, we write $\text{d}\left(q,x\right)<\delta \omega /\left(1-\delta \right)\cdot \text{lfs}\left(x\right)<h_{t}\epsilon^{2}\text{lfs}\left(x\right)$.

With **d**(*q, x*) ≤ 0.55 lfs(*x*), Theorem 12 shows that the normal line from any p∈M intersects $\hat{M}$ exactly once close to the surface. It follows that for every point x∈M with closest point $q\in \hat{M}$, we have $\text{d}\left(x,q\right)\leq \text{d}\left(x,q^{\prime }\right)$ where $q^{\prime }\in \hat{M}$ with *x* its closest point in M. Hence, $\text{d}\left(x,q\right)\leq h_{t}\epsilon^{2}\text{lfs}\left(x\right)$ as well.

Building upon Theorem 12, as a point moves along the normal line at *x*, it is either the case that the distance to $S^{↑}$ is decreasing while the distance to $S^{↓}$ is increasing or the other way around. It follows that these two distances become equal at exactly one point on the Voronoi facet above or below *x* separating some seed $s^{↑}\in S^{↑}$ from another seed $s^{↓}\in S^{↓}$. Hence, the restriction of the mapping *π* to $\hat{M}$ is a homeomorphism.

This shows that $\hat{M}$ and M homeomorphic. Recall that Theorem 4(3) implies U is a *topological thickening* [25] *of* M. In addition, Theorem 3 guarantees that $\hat{M}$ is embedded in the interior of U, such that it separates the two surfaces comprising ∂U. These three properties imply $\hat{M}$ is isotopic to M in U by virtue of Theorem 2.1 in [25]. Finally, as $\hat{M}$ is the boundary of $\hat{O}$ by definition, it follows that $\hat{O}$ is isotopic to O as well.

## 5 Quality guarantees and output size

We establish a number of quality guarantees on the output mesh. The main result is an upper bound on the *fatness* of all Voronoi cell. See Appendix B for the proofs [1].

Recall that fatness is the outradius to inradius ratio, where the outradius is the radius of the smallest enclosing ball, and the inradius is the radius of the largest enclosed ball. The good quality of guide triangles allows us to bound the inradius of Voronoi cells.

**Lemma 14.** *Consider guide triangle t*_*ijk*_*. (1) Edge length ratios are bounded:* $l_{k}/l_{j}\leq \kappa_{l}=\frac{2\delta }{1-\delta }\frac{\sigma \epsilon }{1+\sigma \epsilon }$*. (2) Angles are bounded: $\sin\left(\theta_{i}\right)\geq 1/\left(2{\bar{\varrho }}_{f}\right)$ implying θ*_*i*_ ∈ (7.8°, 165°)*. (3) Altitudes are bounded: the altitude above e is at least α*_*t*_|*e*|*, where $\alpha_{t}=1/4{\bar{\varrho }}_{f}>0.067$.*

Observe that a guide triangle is contained in the Voronoi cell of its seed, even when one of the guides is covered. Hence, the tetrahedron formed by the triangle together with its seed lies inside the cell, and the cell inradius is at least the tetrahedron inradius.

**Lemma 15.** *For seeds* $s_{ijk}\in S^{↑}\cup S^{↓}$, *the inradius of the Voronoi cell is at least $\varrho_{v}\delta \cdot lfs\left(p_{i}\right)$ with* $\varrho_{v}={\hat{h}}_{s}/\left(1+\frac{3}{2\sigma {\bar{\varrho }}_{f}}\right)>0.3$ and ${\hat{h}}_{s}\geq \frac{1}{2}-\left(5+2\eta_{t}\right)\epsilon$.

To get an upper bound on cell outradii, we must first generate seeds interior to O. We consider a simple algorithm for generating $S^{↓↓}$ based on a standard octree over O. For sizing, we extend lfs beyond M, using the point-wise maximal 1-Lipschitz extension $\text{lfs}\left(x\right)=\inf_{p\in M}\left(\text{lfs}\left(p\right)+\text{d}\left(x,p\right)\right)$ [44]. An octree box □ is refined if the length of its diagonal is greater than 2*δ* · lfs(*c*), where *c* is the center of □. After refinement terminates, we add an interior seed at the center of each empty box, and do nothing with boxes containing one or more guide seeds. Applying this scheme, we obtain the following.

**Lemma 16.** *The fatness of interior cells is at most* $\frac{8\sqrt{3}\left(1+\delta \right)}{1-3\delta }<14.1$.

**Lemma 17.** *The fatness of boundary cells is at most* $\frac{4\left(1+\delta \right)}{\left(1-3\delta \right){\left(1-\delta \right)}^{2}\varrho_{v}}<13.65$.

As the integral of lfs^−3^ is bounded over a single cell, it effectively counts the seeds.

**Lemma 18.** $\left|S\right|\leq 18\sqrt{3}/\pi \cdot \epsilon^{-3}\int_{O}lfs^{-3}$.

## 6 Conclusions

We have analyzed an abstract version of the VoroCrust algorithm for volumes bounded by smooth surfaces. We established several guarantees on its output, provided the input samples satisfy certain conditions. In particular, the reconstruction is isotopic to the underlying surface and all 3D Voronoi cells have bounded fatness, i.e., outradius to inradius ratio. The triangular faces of the reconstruction have bounded angles and edge-length ratios, except perhaps in the presence of slivers. In a forthcoming paper [3], we describe the design and implementation of the complete VoroCrust algorithm, which generates conforming Voronoi meshes of realistic models, possibly containing sharp features, and produces samples that follow a natural sizing function and ensure output quality.

For future work, it would be interesting to ensure both guides are uncovered, or both covered. The significance would be that no tetrahedral slivers arise and no Steiner points are introduced. Further, the surface reconstruction would be composed entirely of guide triangles, so it would be easy to show that triangle normals converge to surface normals as sample density increases. Alternatively, where Steiner points are introduced on the surface, it would be helpful to have conditions that guaranteed the triangles containing Steiner points have good quality. In addition, the minimum edge length in a Voronoi cell can be a limiting factor in certain numerical solvers. Post-processing by mesh optimization techniques [5, 53] can help eliminate short Voronoi edges away from the surface. Finally, we expect that the abstract algorithm analyzed in this paper can be extended to higher dimensions.

## Supplementary Material

appendix- supplement

## Footnotes

2 Define $f\left(u,v\right)=\sqrt{1+u^{2}+2uv}-\left(1+u^{2}/2+uv\right)$ and observe that *f*(*u,* −*u/*2) = 0 is the only critical value of *f*(*u, .*). As $\partial^{2}f/\partial v^{2}\leq 0$ for (u,v)∈ℝ×[−1,1], we get that *f*(*u, v*) ≤ 0 in this range.
