# Supplementary material for: Sampling Conditions for Conforming Voronoi Meshing by the VoroCrust Algorithm
Source: Lebniz Int Proc Inform. Author manuscript; Available in PMC 2019 Jan 23. (PMC6344055; doi:10.4230/LIPIcs.SoCG.2018.1)

**A Motivation**

The problem of reconstructing an approximating surface given a finite point sample 𝒫 from an unknown surface ℳ ⊂ ℝ3 is inherently ill-posed as there are, in general, several surfaces that could have produced 𝒫. Therefore, a necessary condition for satisfactory reconstruction is that 𝒫 is dense enough such that all relevant features of the underlying surface can be detected by examining 𝒫 only. The *ε*-sampling framework provides a convenient way to describe such necessary conditions.

We start by recalling that the *medial axis* of ℳ is the closure of the set of points *x* ∈ ℝ3 such that the minimum distance from *x* to ℳ is realized by more than one point on ℳ. This allows us to recall the following core concepts.

**► Definition 27 (Local Feature Size and *ε*-sampling)**

The *local feature size* (lfs) of *x* ∈ ℳ is its Euclidean distance to the medial axis. A sampling is 𝒫 an *ε-sample* if ∀ *x* ∈ ℳ, ‖*x* – *pi*‖ ≤ *ε* lfs(*x*) for some sample *pi* ∈ 𝒫.

This form of sampling was developed for the power crust algorithm [1, 3, 5, 6] and is now standard in the surface reconstruction literature as it provides a convenient theoretical framework to argue that as the sampling density increases, the reconstruction ℳ will converge to the original manifold ℳ. In particular, many reconstruction proofs require that the input samples are an *ε*-sample for *ε* = 0.05 [19] or some other small value, and in practice a larger usually suffices.

Amenta et al. [2,6] established many useful geometric properties of *ε*-samples that apply equally well to our setting. For small *ε*, the manifold locally resembles a planar surface, i.e., the set of samples enclosed in an *ε*lfs-ball are within a few degrees of the tangent plane at the center. In other words, the manifold lies within a tight *cocone*, the complement of a large-angled double cone at the sample [4,20,21].

Many reconstruction methods [3, 9, 19, 28, 31], as in VoroCrust, require samples to meet some density conditions that are qualitatively the same as *ε*-sampling; perhaps relaxed at creases. Additionally, some methods require *sparsity* with a minimum separation between samples, such as a *k*-tight *ε*-sampling where ∃*k* ∈ (0, 1) : ∀*pi*, *pj* ∈ 𝒫, ‖*pi*−*pj*‖ > *k ε* lfs(*pi*) [27]. Our sufficient conditions also require sparsity. Given a dense sampling can convert it into a sparse one and estimate the lfs [30]. For some small and *δ* > *ε*, the union of *δ* lfs-balls

around samples is homotopy equivalent to the manifold [16]. We require a similar local

condition for each sample sphere, and show it is achieved by a sparse sampling.

Homology inference provides an alternative characterization of surface reconstructions from weighted point clouds. As weights are increased from zero, we may track the connectivity the union of balls using persistent homology. This alternative approach has produced several theorems similar to those developed in the *ε*-sampling framework; see, e.g., [12,14,15,18,35].

**Reconstruction Schemes**

Reconstructions based on Delaunay triangulation [13] create primal reconstructions. The power crust [5] is distinguished by its reconstruction being the boundary of some cells of a weighted Voronoi diagram of some constructed seeds. It is closest to our method. The power crust algorithm performs a *primal-dual-dual-primal dance*: compute the dual of the sample points, select a subset as a new set of primal points, weight them, and dualize them to create cells. VoroCrust performs a similar dance, but with key differences in when the weighting is applied, and which Voronoi seeds are selected.

- Power crust uses some far (unweighted) Voronoi vertices of Vor (𝒮) as seeds, to build a (weighted) power diagram whose faces pass through 𝒮.
- VoroCrust uses some nearby points on (weighted) power edges of wVor (𝒮) as seeds, to build a (unweighted) Voronoi diagram whose vertices include 𝒮.

VoroCrust and power crust may produce different vertices, edges, and triangles for the same samples. Indeed, VoroCrust may produce different triangulations by adjusting sample weights. In VoroCrust, all samples are vertices. In power crust, a sample may appear as a vertex, or interior to an edge or facet. Some variations of power crust use filtering to ensure samples are vertices at the price of the reconstruction no longer being the boundary of power cells [1,4,22]. One 2D algorithm uses circles to place Voronoi seeds for segmenting graphics edges around a vertex [24].

Often, the cells of a Voronoi mesh are restricted to the surface [1, 23, 38]. Facets with non- empty intersection are dualized to form the reconstruction facets. Contemporary techniques [25, 36] for Voronoi meshing typically involve creating or moving seeds in a domain, forming their Voronoi cells, then *clipping* the cells by the boundary surface. However, this can lose some important properties such as convexity and connectedness. Alternatively, as in Lloyd iteration to form a centroidal Voronoi tessellation, one may consider getting a stable reconstruction with well-shaped cells from the fixed points of the restricted Delaunay triangulation [29].

**Practical Advantages**

Our companion VoroCrust papers [32,33] demonstrate its practical advantages over other

methods, among other contributions. We summarize both the process advantages and output advantages for motivation. The process advantages are simplicity and robustness. The unweighted Voronoi diagram of the seeds produces the output reconstruction. This can be

generated by established libraries such as Voro++ [37], without modification or special

cases. Clipping requires an implementation of robust constructive solid geometry subtraction

operations, and filtering requires extra geometric checks.

The second advantage is that of the output. VoroCrust cells are true Voronoi cells, with many desirable properties both in theory and applications, such as convexity and fatness, where seeds lie near the medial axis.This locality provides freedom to place additional seeds interior to the volume, away from the surface, without disturbing the reconstruction. For example, additional interior seeds can create a good-quality volume mesh composed of Voronoi polyhedra. These seeds can be placed randomly, or on a structured grid to **B Sandwich Analysis**

The facet separating the two uncovered seeds on an intersection circle is *sandwiched*, lying between the two corresponding guide facets; it also contains the edge dual to the circle.

There are four facets separating a lower and upper guide, the red ones in Figure 4a. These meet at a Voronoi vertex *n* interior to the sliver, sandwiched between the upper and lower triangle pairs. This vertex *n* is a Steiner point of the surface mesh, a vertex that was not a sample. Note that in general *n* is *not* the weighted circumcenter of the sliver, but it does tend to lie close to it when weights are about the same. This “sandwiching” also holds in more general cases of multiple adjoining slivers; see Lemma 30.

We shall see that each sample is still surrounded by a *fan* of facets, meaning the set of facets containing the sample is a topological disk with the sample in its interior.

We first establish some properties about the angular orientation of the fan facets around *p*1, namely that they lie sandwiched between upper and lower guide triangles; their extent is not considered until near the end, at Lemma 30. For orientation, it suffices to consider only the seeds on *S*1, because these are the seeds whose cells contain *p*1, and hence define the fan facet orientations. By excluding other seeds, the fan facets are all triangles extending radially from *p*1 to infinity. This allows us to make the relevant orientation arguments on the surface of *S*1, using the spherical arcs formed by the intersections of extended facets with *S*1. These arcs are great circle arcs between the two points where the two edges bounding the facet intersect *S*1 as they extend from *p*1 to infinity. Later, in Lemma 30, we will clip fan-facets and reintroduce the other seeds.


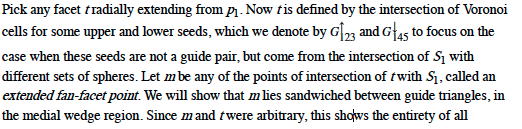


reconstructed triangles are sandwiched. Most of the proofs in this section are technical

arguments about paths on the sphere *S*1 crossing a cap or a crown, as illustrated in Figure 7.


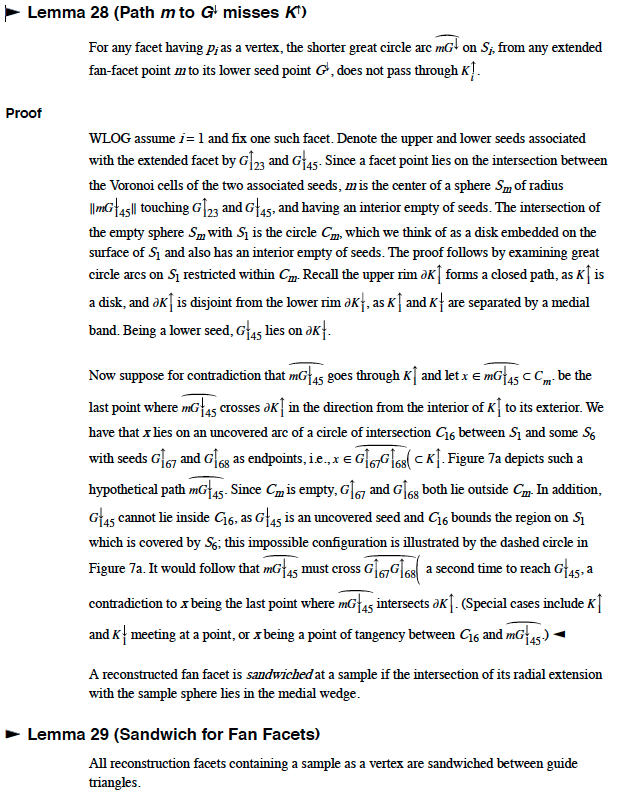


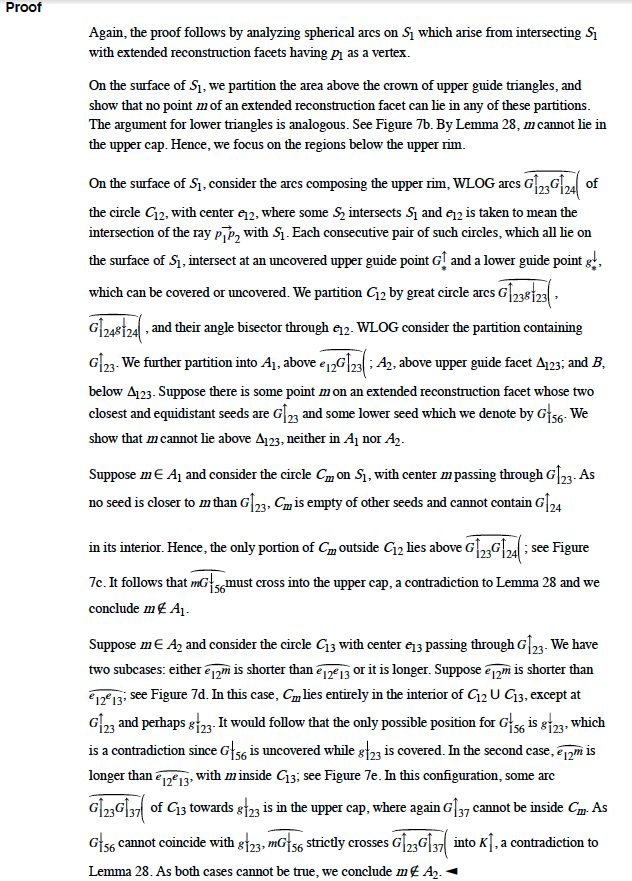


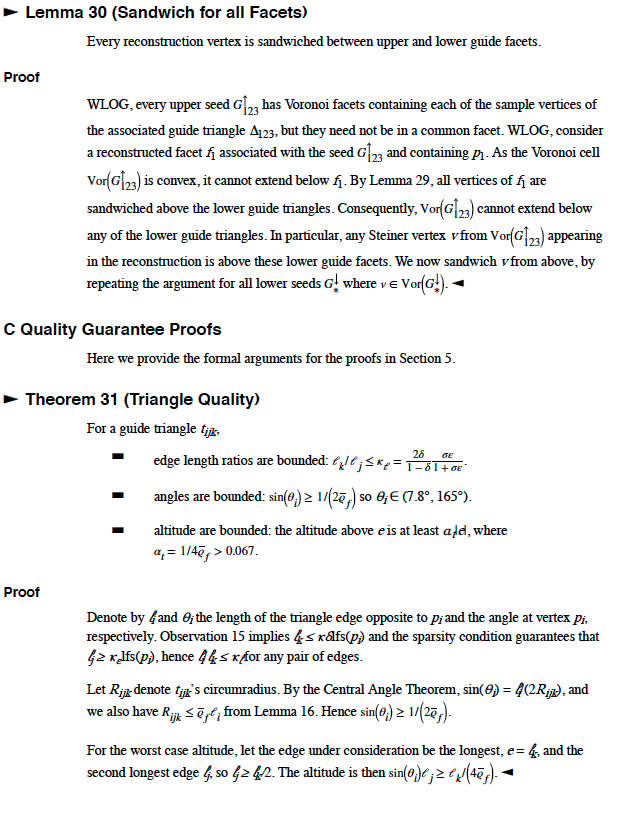


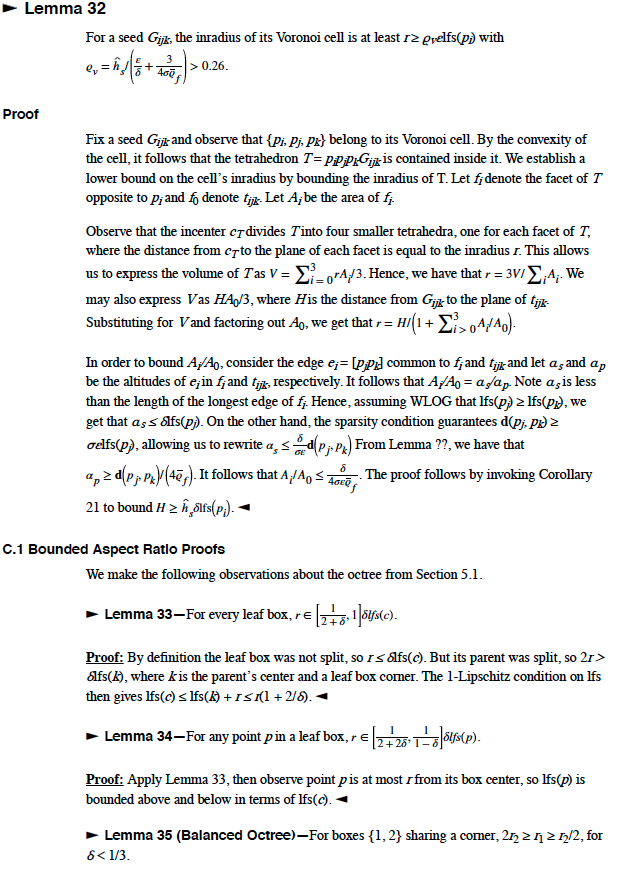


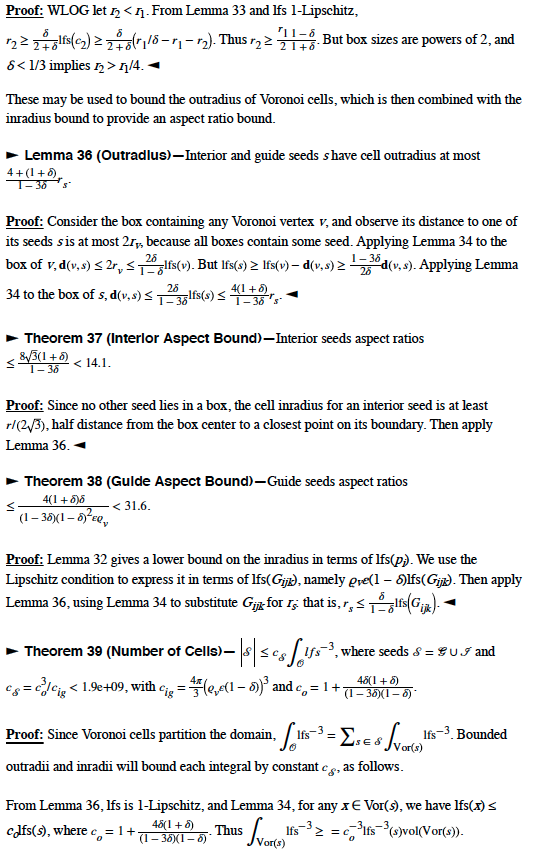


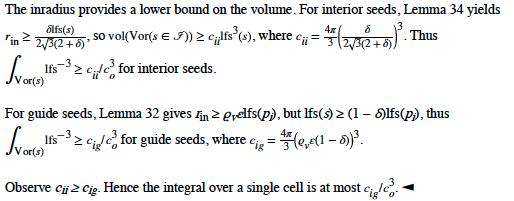

Supplement: appendix- supplement [file NIHMS970243-supplement-appendix-_supplement.docx]
